# Stepwise development of a simulation environment for operating room teams: the example of vertebroplasty

**DOI:** 10.1186/s41077-018-0077-2

**Published:** 2018-09-26

**Authors:** Michael Pfandler, Philipp Stefan, Patrick Wucherer, Marc Lazarovici, Matthias Weigl

**Affiliations:** 1 Institute and Outpatient Clinic for Occupational, Social, and Environmental Medicine, University Hospital, Ludwig-Maximilians-University Munich, Ziemssenstrasse 1, 80336 Munich, Germany; 2 Chair for Computer Aided Medical Procedures & Augmented Reality, Department of Informatics/I-16, TU Munich, Boltzmannstrasse 3, 85748 Garching, Germany; 3 Institute for Emergency Medicine and Management in Medicine (INM), University Hospital, Ludwig-Maximilians-University Munich, Schillerstraße 53, 80336 Munich, Germany

**Keywords:** Cognitive task analysis, Non-technical skills, Observation, Interview, Multidisciplinary, Vertebroplasty

## Abstract

**Background:**

Despite the growing importance of medical simulation in education, there is limited guidance available on how to develop medical simulation environments, particularly with regard to technical and non-technical skills as well as to multidisciplinary operating room (OR) team training. We introduce a cognitive task analysis (CTA) approach consisting of interviews, structured observations, and expert consensus to systematically elicit information for medical simulator development. Specifically, our objective was to introduce a guideline for development and application of a modified CTA to obtain task demands of surgical procedures for all three OR professions with comprehensive definitions of OR teams’ technical and non-technical skills.

**Methods:**

To demonstrate our methodological approach, we applied it in vertebroplasty, a minimally invasive spine procedure. We used a CTA consisting of document reviews, in situ OR observations, expert interviews, and an expert consensus panel. Interviews included five surgeons, four OR nurses, and four anesthetists. Ten procedures were observed. Data collection was carried out in five OR theaters in Germany.

**Results:**

After compiling data from interviews and observations, we identified 6 procedural steps with 21 sub-steps for surgeons, 20 sub-steps for nurses, and 22 sub-steps for anesthetists. Additionally, we obtained information on 16 predefined categories of intra-operative skills and requirements for all three OR professions. Finally, simulation requirements for intra-operative demands were derived and specified in the expert panel.

**Conclusions:**

Our CTA approach is a feasible and effective way to elicit information on intra-operative demands and to define requirements of medical team simulation. Our approach contributes as a guideline to future endeavors developing simulation training of technical and non-technical skills for multidisciplinary OR teams.

**Electronic supplementary material:**

The online version of this article (10.1186/s41077-018-0077-2) contains supplementary material, which is available to authorized users.

## Background

Although high fidelity simulation is widely adopted in medical education, there is only limited guidance on simulator development [1]. With regard to medical simulation, it is essential to gather precise information on the actual procedure prior to the development of a simulation environment. Moreover, information on operating room (OR) team coordination, interaction, and communication are highly relevant to surgery and significantly broaden the application of simulation [2]. This is in line with the call for medical training environments that encompass non-technical skills (NTS) training, facilitate collaborative learning, and assess multidisciplinary care teams [3, 4]. Systematic guidance on simulator development and consensus-based definition of simulation requirements are therefore needed.

### Multidisciplinary OR team simulation training

Currently, the majority of simulation training in surgery is designed to train technical skills (TS) of individual participants [5]. In contrast, adverse events in the OR are often associated with erroneous or missing non-technical skills (NTS) [6, 7]. NTS in the OR are defined as “the cognitive and social abilities that complement surgeons’ technical expertise, clinical knowledge, and procedural skills in the operating room (OR)” [8 p.1124]. Although various definitions have been applied, we particularly consider communication, situation awareness, decision-making, teamwork, and leadership to be key NTS [6]. NTS are essential for surgical success, procedure efficiency, and patient outcomes [7].

NTS training as well as TS training can be incorporated into OR multidisciplinary team training, which has been defined as training at least one participant from surgery, nursing, and anesthesia simultaneously [9]. But few studies meet these criteria [4, 9], and actual OR teams often lack effective NTS [7, 10]. Therefore, training of the entire OR teams on TS and NTS is important as teams that work together should also train together [11]. Nevertheless, to the best of our knowledge, there are no methods established for developing medical simulations for multidisciplinary OR teams. We therefore introduce a systematic approach for stepwise development of simulation environments for surgical teams.

### Cognitive task analysis for identification of simulator requirements

Procedural simulators for individual trainees in healthcare are mostly based on cognitive task analysis (CTA). CTA has been introduced to reliably elicit information on cognitive regulation and task performance [12], as modern work practices in complex sociotechnical work systems involve unobservable tasks like decision-making, planning, and problem-solving [1]. CTA identifies cognitive aspects of expertise from subject matter experts (SMEs) and was developed for “… identifying, analyzing, and structuring the knowledge and skills experts apply when they perform complex tasks” [13 p.541]. As medical experts tend to omit information when describing a task, CTA reduces the risk of missing or failing information [14, 15]. Simulation training in medical education that is based on CTA methods has various advantages: it is efficient, adheres to participants’ needs, and is associated with superior training outcomes compared with traditional methods of medical training [16, 17]. Moreover, CTA-based training saves training time [18].

CTA is not one particular method, but a set of different approaches with more than 100 types of CTAs available [13, 19]. Referring to Yates [20], structured or semi-structured interviews are the most frequently used CTA method. Interview-based CTAs should include at least three to four SMEs to capture the full amount of knowledge [21]. SMEs develop a connection between the important cues of the clinical procedure and appropriate behavioral responses [22]. For development of medical simulators, CTA-based interviews need to identify these cues [12, 23].

In situ observations are a powerful and frequently used approach to elicit knowledge [12]. They help to scrutinize tasks being performed, identify interactions among team members like communication and coordination, and help to elicit demands and processes, strategies of skilled workers, and how work is done [19, 22]. On-site observations complement other CTA approaches, such as interviews, to establish a comprehensive understanding of the task.

Researchers developing a simulation environment need a thorough understanding of the task and its key characteristics [24]. Therefore a team consisting of clinicians, simulator developers, engineers, and human factor experts should collaboratively deduce and define key characteristics of the simulator environment. Their decisions should be derived from observational and interview data in order to ensure comprehensive consideration of the medical procedure, intra-operative requirements, technical feasibilities, and resources.

## Objectives

We sought to introduce a CTA approach that can be used as a guideline on how to elicit and represent knowledge from experts to develop a multidisciplinary team simulation environment for surgical training of technical and non-technical skills. Specifically, we aimed:

1. To define all steps and sub-steps of a surgical procedure; and
2. To identify intra-operative technical and non-technical skills relevant to all involved OR professions, i.e., surgeons, nurses, and anesthetists for multidisciplinary team training; and
3. To analyze the results collaboratively with clinicians, computer scientists, and human factor experts to deduce the simulation requirements.

## Methods

Before starting the study, we obtained ethical approval from the Ethics Committee of the Faculty of Medicine, LMU University (Nr 773-15). All participants were informed about the purpose of the study and confidentiality of data and provided written consent prior to data collection.

To demonstrate our methodological approach, we use vertebroplasty (VP) as an example. VP is a percutaneous, minimally invasive procedure where a needle (trocar) is inserted into a fractured vertebra under C-Arm or CT guidance to inject bone cement for stabilization. It is either performed one-sided or two-sided, i.e., inserting trocars through one or both pedicles of the vertebra. Main target group is patients with osteoporotic compression fractures. Usually, this procedure is carried out in roughly half an hour by an orthopedic, trauma, or neuro surgeon. The OR team further consists of one anesthetist and at least one sterile nurse. VP is widely applied and carried out by different specialties and is therefore suitable for medical simulation training where all team members are trained together on TS and NTS.

Our CTA-based approach that encompassed in situ observations in the OR, expert interviews, and an expert consensus panel consisted of two parts: knowledge elicitation and knowledge representation [24]. For the first part, elicitation aims to capture experts’ cognitive knowledge that is often tacit and automated [1]. As second part, knowledge representation means the subsequent interpretation, description, and representation of the elicited knowledge [22]. It is recommend to conduct knowledge elicitation and representation in five steps [22, 25]: collect preliminary knowledge (step I), identify knowledge representations (step II), and apply focused knowledge elicitation methods (step III). Steps I–III form knowledge elicitation. Knowledge representation consists of analyzing and verifying data acquired (step IV) and formatting results for intended application (step V). Table 1 depicts these steps and our methods.

**Table 1:** Overview of CTA parts, steps, and methods applied

Cognitive task analysis					
CTA parts	Knowledge elicitation			Knowledge representation	
Steps	Step I	Step II	Step III	Step IV	Step V
What was done? (Aims)	Collect preliminary knowledge	Identify knowledge representations	Apply focused knowledge elicitation methods	Analyze and verify data acquired	Format results for the intended application
How was it done? (Methods)	Procedure descriptions, case reports, and video tapes	Identify key contents of the medical procedure	OR observations and expert interviews	Evaluation of observations and interviews and establish consensus	Format data and create tables

### Steps I and II: collection of preliminary knowledge and identification of knowledge representations

The objective of step I was to collect preliminary knowledge on the target procedure, i.e., vertebroplasty. We studied procedure descriptions and case reports and reviewed available video tapes. Procedure descriptions and case reports were retrieved from surgical handbooks and journals. Video tapes were obtained from Internet video portals. For step II, we identified key tasks of the procedure and respective knowledge representations. Hereby, we used information collected during step I and examined each task to identify subtasks and types of knowledge required to perform this task. On the team level, we surveyed for dependencies between surgeons, nurses, and anesthetists.

In preparation of step III, we first determined appropriate methods to elicit knowledge from experts. To decide on CTA methods, it is important to consider which types of cognitive processes and tasks should be investigated [24]. We defined requirements of the CTA methods to obtain essential information on tasks and simulation requirements. Specifically, not only information on task sequences, demands, and equipment were essential, but also information on decisions, sensory experience, and teamwork was deemed relevant. As different CTA techniques may represent different aspects of knowledge, it is recommended to combine two or more methods [19]. Based on established CTA literature [e.g., 12, 19, 22], we decided on semi-structured observations and semi-structured interviews. Observations in naturalistic OR settings allow to compare an expert’s description of a task with actual events. Interviews are the most direct way to elicit experts’ knowledge with the appropriate depth. Our complementary combination of observational and self-report methods is consistent with previous recommendations on CTA [1, 13, 19] and has been previously applied in CTA-based case studies in medical settings [26].

### Step III: application of focused knowledge elicitation methods

Prior to observations, we defined aims and important aspects of this third step. The purpose of in situ observations using semi-structured data collection was (1) to develop a deeper understanding of the VP procedure in real-world ORs and determine its main procedural steps, (2) to observe team roles and teamwork behaviors as well as intra-team communication and coordination (including leadership), and (3) to identify potential deviations in intraoperative practice in course of VP procedures. With these purposes in mind, we developed an observational guideline. The guideline can be found as Additional file 1 (A1 “Observation guideline”). Observations were conducted in the OR by two experienced and trained observers (MP, MW). After the procedure, both observers reviewed their notes and their results were cumulated for final evaluation.

The aim of the semi-structured interviews was to gather information on VP for a detailed and thorough understanding of its technical and non-technical tasks, steps, and key behaviors. We decided on the Concepts-Processes-and-Principles-Method [27, 28] because it is considered as an evidence-based CTA method [29]. It elicits knowledge based on predefined categories and has been used successfully in medical settings [30]. We modified the categories for our purpose (development of simulation environment for OR teams including TS and NTS) after our preliminary collection of knowledge (steps I and II). For example, we added the categories communication and coordination to elicit information on interactions and dependencies between team members. A description of all included categories can be found in Table 3. A comparison between the original [12] and our modified categories can be found as Additional file 2 (A2 “Category comparison”). According to the Concepts-Processes-and-Principles approach, tasks are divided into steps (phases) and sub-steps, while at least three to four SMEs have to be asked the same questions for every sub-step [21]. We deduced all steps and sub-steps of a VP from procedure descriptions, our observations, and a pilot interview with an expert. If a step could not be divided into any more sub-steps by the expert, the next step starts. We decided to start our simulation with the step “intra-operative preparation” and end it with the completion of the “closure” step since these include all intra-operative behaviors and activities for our simulation purposes. For the steps before and after, we only gathered basic information. Interview guidelines including all relevant categories were designed for all three OR professions respectively. As an example, the surgeon’s interview guideline can be found as Additional file 3 (A3 “Surgeons interview guideline”). During each interview, the SMEs were asked questions pertaining to each sub-step of the procedure. To the best of our knowledge, there is no definite criterion for who is considered SME. In the literature, various criteria such as numbers of procedures and years of experience can be found [31]. Moreover, the validity of selection criteria has been discussed, e.g., for surgeons, the number of successful procedures instead of the overall count of operations should be considered [27]. Therefore, we decided to recruit SMEs that are considered and suggested as experts in their field by their colleagues. A convenience recruitment strategy was applied, i.e., senior surgeons (in the field of neurosurgery, trauma, and orthopedics) in various hospitals were directly asked for an interview. Additionally, we asked observed professionals for suggestions of potential SMEs. All interviews were recorded on a voice recorder and transcribed verbatim afterwards.

### Steps IV and V: analysis, verification of data, and formatting of results for intended application

The aim of step IV was to evaluate observation and interview results to establish simulation experts’ consensus on what is considered essential for implementation in the simulation environment. We used Protocol Analysis [32] for this step that classifies data into pre-defined categories and for an intended purpose, i.e., simulation development. Although alternative methods to analyze data are available (see [20] for more information), we decided on this approach as we already used these pre-defined categories in step III.

We started by analyzing our observational data. All observation notes were jointly reviewed, and information was classified in a table for surgeons, anesthetists, and nurses respectively. These tables consisted of the categories (table columns) and sub-steps (table rows) of the interview guidelines. Then, we coded and categorized all transcripts of the anonymized interviews and transferred the results into the three tables. In the next step, our team discussed all three result tables to establish a consensus on respective simulation requirements. Our expert team of simulation developers and behavioral analysists consisted of two psychologists (MP, MW), two computer scientists with experience in simulator development (PS, PW), and a medical doctor (ML) with a computer science degree and extensive experience in medical simulation training. Together, the panel examined every category of each sub-step in the three result tables and identified important tasks, cues, and other relevant information to be implemented into the simulation environment. Our resulting findings were classified into the newly formed category “simulator requirements.”

The aim of step V was to format all results for final application. The Concepts-Processes-and-Principles-Method combined with Protocol Analysis already generates a detailed list and description of the actions, conceptual knowledge, and conditions necessary to perform a task (see Additional file 4 [A4 “Result Tables”] for comprehensive result tables). Additionally, results were revised and streamlined for clarity and comprehensibility. We created a table with the steps and sub-steps for all three OR team professions (Table 2). For demonstration purposes, we additionally compiled a table with all analyzed categories for a single sub-step for all three OR professions respectively (Table 3).

**Table 2:**
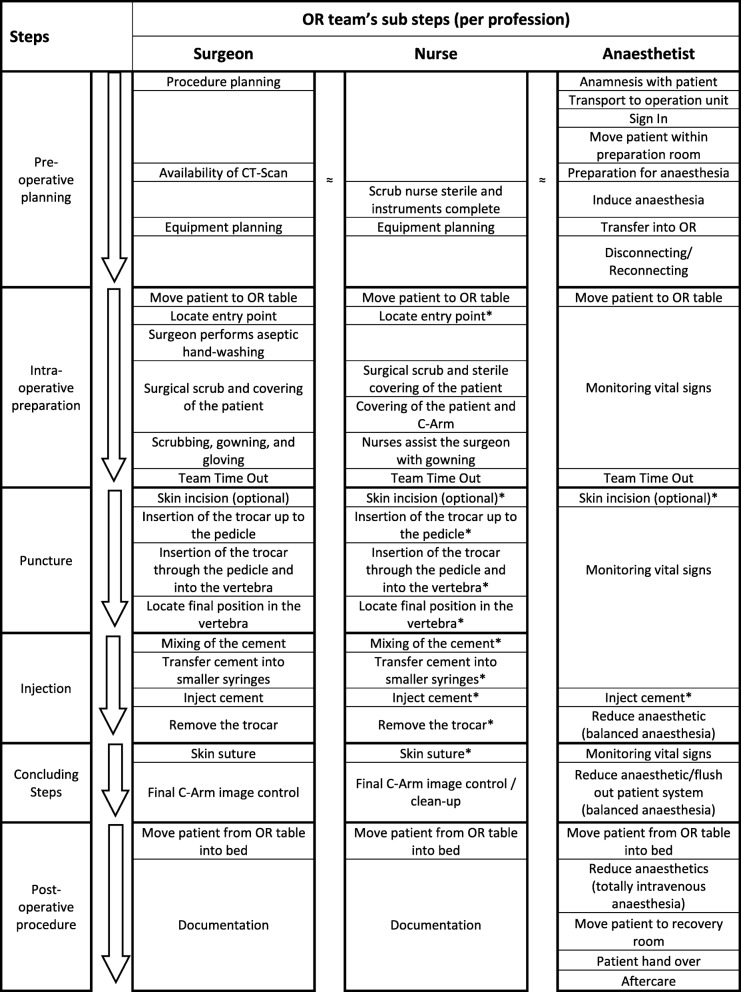
Steps and sub steps of a vertebroplasty for surgeon, nurse, and anesthetist

*We used identical terms for the shared sub-steps for simplification; the tasks on hand of the single professions differ. See Additional file 4 (A4 “Result tables”) for more information on the tasks

**Table 3:** Intra-operative demands for surgeon, nurse, and anesthetist (example sub-step “inject cement”)

Categories	Profession		
	Surgeon	Nurse	Anesthetist
Objective Objectives for this sub-step?	Inject cement evenly and adequately to stabilize fractured vertebral body	Assist surgeon to inject cement	Ensure patient safety
Party responsible Who is responsible?	Surgeon	Surgeon	Anesthetist
Course of action How do you proceed?	Inject cement slowly under lateral C-Arm guidance	Hand over injection system to surgeon; provide feedback on cement’s time status	Monitoring vital signs
Decisions What decisions do you need to take?	(1) When to apply cement (2) Amount of cement to inject (3) Pressure and speed of injection	(1) Can cement be applied (2) How long can cement be applied	(1) Increase oxygen saturation
Basis for decisions On what base do you take these decisions?	(1) Time since cement was mixed, tactile cement probing (like “chewing gum”), experience; (2) Volume of vertebral body, type of fracture; (3) Leakages, experience	(1) Cement should “curl” instead of falling down (2) Depends on type, temperature, and mixing container	(1) Existing risk factors
Attention Focus of attention?	C-arm guidance, cement amount and flow direction, fracture line	Time	Vital signs; signs of reactions to cement
Information Important information?	X-ray picture, injected amount (in mm^2^) on syringe	–	Pitch of oxygen saturation
Feedback What feedback do you get?	No haptic feedback through injection, vital signs from anesthetist	–	–
Equipment Tools and equipment in use?	Syringe or filler, trocar, C-arm, 2nd monitor	Applicators or syringes	Monitoring devices (ECG, blood pressure, oxygen saturation, temperature)
Communication What communication is necessary?	To anesthetist that cement injection starts, to circulating nurse to reposition C-arm, to scrub nurse how long since cement has been mixed	Scrub nurse asks what material is needed for the step “cutaneous suture” (if not already arranged)	To surgeon if vital signs change significantly
Coordination What coordination takes place?	Handing of syringe from scrub nurse to surgeon	Handing of syringe from surgeon to scrub nurse; empty syringes into waste	If surgeon needs longer, anesthetist may give medication that supports circulation
Time-sensitive Is this sub step time-sensitive?	Yes, as cement can only be injected within a limited duration	Yes, cement hardening needs to be monitored	–
Importance/patient risks Is this a high-risk sub step?	Critical phase with higher patient risks	Higher risk	Critical phase with higher patient risks
Automated action Is this action automated?	Non-automated action	Time has to be monitored actively	–
Potential complications What kind of complications could occur?	Cement leakage into vessels, spinal canal, or intervertebral disk; too much injected cement; pulmonary embolism	–	Blood pressure may fall if surgical stimulus is missing for too long
Variations Are there any variations to your approach?	Different types of cement, different cement injection systems	–	–

## Results

### Observational and interview sample

Data collection was conducted in five hospitals in Germany and Austria between December 2015 and June 2016. As VPs are performed by different medical specialties, we observed ten VPs at two neurosurgery departments, two orthopedics departments, and one trauma surgery department with a mean duration of 29.6 min and a range from 15 to 60 min. Of those ten VPs, two were one-sided and eight were two-sided, eight used one C-Arm and two used two C-Arms. All procedures were elective and were performed under general anesthesia. All teams had previous experience working with each other of approximately 3 years.

Expert interviews were conducted with five surgeons, four nurses, and four anesthetists with mean durations of 47, 61, and 56 min respectively. All five surgeons were male with two being neurosurgery specialists, two trauma specialists, and one orthopedic specialist. All had at least 10 years of experience with VPs. Of the four nurses, two were female. All had at least 7 years of professional tenure. All four anesthetists were male, and all but one had at least 15 years of experience. This one was about to successfully complete his specialty training and was explicitly suggested as expert with regard to our criteria by his colleagues and head of department. All interviewees successfully completed more than 100 VPs and can therefore be considered experts.

### CTA results

We present the results as an overview according to our three study objectives. Specific examples from the target procedure are used as to demonstrate the findings. Due to brevity of space, in-depth presentation of all retrieved data is provided as Additional file 4 (A4 “Result Tables”).

1. Definition of steps and sub-steps of the procedure

We identified 6 procedural steps with 21 sub-steps for surgeons, 20 sub-steps for nurses, and 22 sub-steps for anesthetists (see Table 2 for an overview and Additional file 4 [A4 “Result tables”] for an in-depth presentation). As can be seen in Table 2, the surgeons’ sub-steps differ from the anesthetists’ sub-steps, whereas surgeons’ and scrub nurses’ sub-steps are almost identical. For example, surgeons’ task steps from “locate entry point” till “transfer cement into smaller syringes” concur with anesthetists’ task of “monitoring vital signs,” except for the steps “team time out” and “skin incision.” Surgeons and nurses share 18 of their 21 respectively 20 sub-steps while anesthetists only share 5 sub-steps with either of them. However, although we consistently used the identical terms for the shared sub-steps for simplification, the tasks on hand of the single professions differ. As an example, the sub-step “inject cement” is presented in detail in the next section and in Table 3. Interactions and dependencies between single professions can be seen in the categories “communication” and “coordination.” Additionally, we identified considerable variation in OR teams’ sub-steps. For example, there are varying anesthesia procedures possible, depending on the type of anesthetic used, and therefore, the sub-steps change accordingly. Moreover, in the step “preoperative preparation,” there are fewer overlapping sub-steps for all OR members due to their individual tasks while preparing and setting up.

2. Definition of intra-operative technical and non-technical skills relevant to all involved OR professions

We collected information on 16 predefined categories for all three OR professions through observations and interviews. Variations in the procedure are possible due to different hospitals and their particular standards, different departments (neurosurgery, trauma, and orthopedics), and available equipment (one or two C-Arms, CT). Table 3 provides an example excerpt for the sub-step “inject cement” that lists intra-operative demands for each OR profession. We chose this sub-step because it bears high patient risks, and all three OR team members need to collaborate closely and pay particular attention to each other’s actions. The full results reporting the comprehensive descriptions of all OR professionals’ intra-operative task demands can be found as Additional file 4 (A4 “Result Tables”).

3. Consensus-based deduction of simulation requirements

**Fig. 1:**
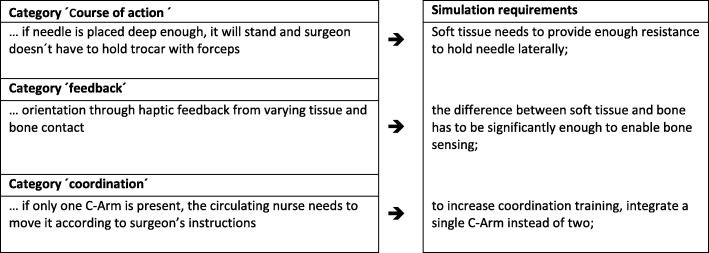
Intra-operative demands and simulation requirements for surgeons for the task step ‘place trocar on pedicle’

All task information was then discussed in an expert panel to achieve consensus on simulation requirements of intra-operative demands. The comprehensive list of defined requirements for the whole procedure can be found as Additional file 4 (A4 “Result Tables”). Figure 1 represents the consensus on team’s task step “place trocar on pedicle” as an example. On the left side of Fig. 1, the previously established intra-operative demands are listed; in the case of this example of surgeons’ sub-step “insertion of the trocar up to the pedicle,” the expert panel deduced respective simulation requirements (see right column, Fig. 1). Here, surgeons reported that a needle placed on the pedicle is able to stand independently if it is inserted deeply enough into the soft tissue (cf., category “course of action”). Accordingly, simulation requirements were defined such that soft tissues of the simulator provide sufficient resistance to laterally hold the needle. Moreover, surgeons reported that during initial trocar placement, they rely upon haptic feedback from varying tissue and bone contact for orientation (cf., category ‘feedback’). Consequently, the difference between soft tissue and bone needs to be noticeable for sensing bone surface and structure. Additionally, surgeons revealed that the absence of a second C-Arm leads to more coordination needs between surgeon and circulating nurse, as the single C-Arm needs to be turned by the circulating nurse on the surgeon’s instructions constantly (cf., category “coordination”). Therefore, to increase coordination training, a simulation environment may deliberately implement a single C-Arm instead of two.

## Discussion

We introduce a CTA approach that may serve as a guideline for team simulation environment development. It contributes in several ways to the current knowledge base on simulation in surgery:

First, we show how to identify tasks and demands of a surgical procedure that provides a basis for definition of realistic simulation environments. In comparison with existing studies on simulator development [33, 34], our approach allows generation of information about the procedure, inherent demands and skills, and simulator environment characteristics that emerge from the actual surgical procedure. Our approach thus combines the instructiveness of CTA-based methods with the explorative nature of simulation development [19, 35]. We are convinced that this systematic development process facilitates the construction of surgical simulation environments that enable “the operator to reproduce or represent under test conditions phenomena likely to occur in actual performance” [36].

The definition and simulation of key tasks and learning objectives are considered as being more important than fidelity in simulation [37, 38]. The systematic combination of structured observations and expert interviews generates reliable information of the surgical procedure’s key tasks through minimizing biases and sources of unreliable information that are inherent to each of the individual methods. By using different methods, single sources of bias like recall bias in SME-interviews or observer bias in realistic observations are minimized. Potential bias and erroneous information are compensated through the combination of different methodologies. Clearly defined key tasks and learning objectives are prior conditions for the design of training technologies [39] that have a sustainable impact for the transfer of technical skills from simulation to patient outcomes [5]. Our approach strengthens the evidence of validity of the findings and provides a plausible basis for the design of a simulation environment.

Moreover, our approach captures technical and non-technical demands relevant for all OR professionals, i.e., surgeons, nurses, and anesthetists. This perspective of OR teamwork provides basis for future development of multidisciplinary OR team training [9]. Current simulation literature in this field frequently lacks descriptions about the development of medical team training and scenarios [40]. Our method facilitates definition of surgical OR team simulations that include all essential behaviors, skills, key demands, and intra-operative steps. We are confident that our systematic definition procedure, through precise development description and elicitation categories, can be used for other medical procedures, beyond spine or minimally invasive surgery. A wider adaption of this method would limit previous criticism that available NTS and multidisciplinary OR team trainings often lack thorough reporting of their development process [4, 41].

A further contribution of our work relates specifically to the field of simulation in spine surgery. Simulators in spine surgery are increasingly introduced since many procedures involve high risks [3, 42]. While other surgical specialties already incorporate team training in their assessment and education, there are no team simulations available for spine procedures. Despite the introduction of simulators for minimally invasive spine surgery [3, 43], concurrent reports omit information on how the simulator protocol was actually obtained and how simulator requirements were deduced. Our attempt to take account of NTS across all involved professional roles in the OR expands previous work since team simulations are key to surgical safety and quality of care [3]. This concurrent inclusion of all OR professions prevents silo development and separation of professions in improvement efforts of OR teamwork [44].

### Limitations

Although we successfully applied our CTA-based approach for the vertebroplasty procedure and OR teamwork, the external validity of our results may be limited concerning other surgical procedures, e.g., OR teams in more complex procedures and consisting of additional OR professionals. Our approach was well suited for VP and minimally invasive surgery, characterized by small teams, short durations, few risk-associated steps, and a homogeneous patient group. Other surgical procedures may require a more adaptive approach, e.g., consideration of task scheduling. Wei and Salvendy [19] offer an extensive discussion of other types of CTAs and their possibilities. We sought to minimize bias by utilizing different complementary methods. Yet, we cannot rule out information bias since observers, interviewers, and part of the reviewers of step IV and V were identical (MP and MW).

### Experience reflection on potential benefits, challenges, and recommendations

To guide other researchers utilizing CTA for surgical simulation development, we report information on our experience and discuss our benefits, challenges, and recommendations conducting a CTA.

Concerning potential benefits, this CTA approach generated an in-depth understanding of the surgical procedure and all its characteristics, challenges, and processes. We were able to gather information and insights about procedure characteristics that are not reported in textbooks, training lessons, or taught by senior experts. This representation of intra-operative demands for all OR team members during all steps of a vertebroplasty will guide the further development of our simulation environment.

One of the biggest challenges for us was to select the appropriate CTA methods for our aims, as there are many CTA methods available and only limited literature on their pros and cons exists. Moreover, similar development studies do not describe their methods and underlying choices in depth. We therefore suggest, prior to start, a careful literature review and consultation of a CTA expert. Additionally, the nomenclature within the CTA literature can be confusing. For example, the second part of a CTA is labeled “knowledge representation” and can be mistaken for step II “identify knowledge representations” which belongs to the first part of CTA (see Table 1). Although this can be misleading, we decided not to change nomenclature for enhanced comparability with other CTA literature. A further challenge was the complexity of CTA approaches: they are time-consuming and require a set of skills like accuracy, endurance, patience, and understanding of complex tasks. Experts are often hard to recruit for interviews that last longer than 30 min. During CTA interviews, we experienced that surgical experts perceive it as challenging to unravel a complex procedure in such details, i.e., fine-grained tasks steps with repeatedly explaining decisions and demands.

Our recommendations are therefore to carefully choose the appropriate CTA method as the results will strongly depend upon the selected approach. Our method was feasible for defining VP simulation development demands, but other scenarios may require different or additional CTA methods. Moreover, it might be necessary to adapt or modify CTA methods to specific needs and aims. Additionally, we recommend to include a surgical expert or specialist in the development team from the very start (i.e., behavioral analysts in general lack surgical insights). Moreover, surgical specialists on the team can help recruiting other experts for interviews and observations. In addition, we recommend conducting pilot observations for adapting to surgical context and collecting preliminary knowledge (i.e., to provide a “feeling” for the surgical procedure). Lastly, we recommend identifying and contacting experts as soon as possible as recruiting can be time-consuming due to their rotations and acutely changing schedules.

### Implications for future studies and simulation practice

This approach may guide future studies aiming at structured and systematic identification and definition of TS and NTS in the course of surgical simulator development. Adaptions to other surgical procedures in and outside minimally invasive and spine surgery and to other procedure characteristics, e.g., large teams or interchangeable procedure steps, are recommended. Through identification of simulation demands and reporting of the development process, our approach may facilitate future endeavors to bridge the gap between a medical procedure and its simulation. Recent systematic reviews showed an overall low methodical quality for medical simulation studies with weak standardized reporting of the development [3, 9]. In particular, knowledge elicitation and representation methods need to be reported thoroughly with the objective to establish reliable and valid simulation environments for education and training. Future development of medical training simulators needs to draw upon systematic and well-structured methods of task and skill identification as well as concise definition of simulator requirements with comprehensive reporting. Medical skill training based on CTA approaches is recommended.

## Conclusions

Through a CTA approach using in situ observations and semi-structured interviews and protocol analysis, we defined steps, sub-steps, and demands of vertebroplasty procedures for all three OR professions and deduced simulation requirements in an expert panel. In our experience, CTA is a well-suited and effective method for simulator development to elicit, interpret, and represent information on intra-operative demands. This work contributes to future approaches, guiding them on implementing training for multidisciplinary OR teams regarding TS and NTS.

## Additional files

Additional file 1: A1 “Observation guideline.” (PDF 143 kb)

Additional file 2: A2 “Category comparison.” (PDF 337 kb)

Additional file 3: A3 “Surgeons interview guideline.” (PDF 243 kb)

Additional file 4: A4 “Result Tables.” (XLSX 55 kb)

## Abbreviations

CTA: Cognitive task analysis
NTS: Non-technical skills
OR: Operating room
SME: Subject matter expert
TS: Technical skills
VP: Vertebroplasty
