# Supplementary material for: Stepwise development of a simulation environment for operating room teams: the example of vertebroplasty
Source: Adv Simul (Lond). 2018 Sep 26;3:18. doi: 10.1186/s41077-018-0077-2 (PMC6158852; doi:10.1186/s41077-018-0077-2)
Supplement: Supplementary file 1 — A1 “Observation guideline.” (PDF 143 kb) [file 41077_2018_77_MOESM1_ESM.pdf]

Observation Guideline

Location: Date: Start: End:

Observer: Observation Number:

OR Team Members:

- Surgeon 1 (S1)
- Surgeon 2 (S2)
- Nurse 1 (N1)
- Nurse 2 (N2)
- Anaesthetist 1 (A1)
- Anaesthetist 2 (A2)
- Other:

Procedure: Procedure details:

Observational Dimensions:

Procedure-Phase: what is happening, what is being done?

Communication: Sender, recipient, form of communication (verbal, non-verbal; question, answer), content (informative, guiding, private), (assumed) purpose…

Coordination: who, where, where to, what for, interdependency…

Procedure-specific observations: alterations, decisions, tools, OR team members, constraints…

Procedure-Phase	Observational Dimensions		
	Communication	Coordination	Procedure specific observations

Observation Number:
Page:
