# Supplementary material for: Stepwise development of a simulation environment for operating room teams: the example of vertebroplasty
Source: Adv Simul (Lond). 2018 Sep 26;3:18. doi: 10.1186/s41077-018-0077-2 (PMC6158852; doi:10.1186/s41077-018-0077-2)
Supplement: Supplementary file 2 — A2 “Category comparison.” (PDF 337 kb) [file 41077_2018_77_MOESM2_ESM.pdf]

Comparison of the original and modified Categories of the Concepts-Process-and-Principles-Method

Original Categories	Modified Categories
• The sequence of actions (or steps) necessary to complete the subtask	• Course of Action<br>How do you proceed?
• The decisions that have to be made to complete the subtask, when each must be made,	• Decisions<br>What decisions do you need to take?
• the alternatives to consider,	• Variations<br>Are there any variations to your approach?
• and the criteria used to decide among the alternatives	• Basis for decisions<br>On what base do you take these decisions?
• All concepts, processes, and principles that are the conceptual basis for the experts' approach to the subtask	• Objective<br>Objectives for this sub step?<br>• Attention<br>Focus of attention?<br>• Information<br>Important information?<br>• Automated Action<br>Is this action automated?<br>• Potential complications<br>What kind of complications could occur?
• The conditions or initiating events that must occur to start the correct procedure	
• The equipment and materials required	• Equipment<br>Tools and equipment in use?
• The sensory experiences required (e.g., the analyst asks if the expert must smell, taste, or touch something in addition to seeing or hearing cues in order to perform each subtask)	• Feedback<br>What feedback do you get?
• The performance standards required, such as speed, accuracy, or quality indicators	• Time-sensitive<br>Is this sub step time- sensitive?<br>• Importance/Patient Risks<br>Is this a high risk sub step?
	• Party Responsible<br>Who is responsible?<br>• Communication<br>What communication is necessary?<br>• Coordination<br>What coordination takes place?
