# Supplementary material for: Stepwise development of a simulation environment for operating room teams: the example of vertebroplasty
Source: Adv Simul (Lond). 2018 Sep 26;3:18. doi: 10.1186/s41077-018-0077-2 (PMC6158852; doi:10.1186/s41077-018-0077-2)
Supplement: Supplementary file 3 — A3 “Surgeons interview guideline.” (PDF 243 kb) [file 41077_2018_77_MOESM3_ESM.pdf]

Guidelines for SME Interviews

Cognitive Task Analysis of the surgical procedure (on individual and team level)

Explanatory Introduction:

Dear Mr./Ms./Mrs. Name thank you very much for your time and for helping our research project and us. As we already told you, we are working on the development of a virtual-reality simulator for vertebroplasty (VP). Therefore, I want to talk through an entire VP procedure step by step with you. This will give us important information about the procedure, which we will then integrate into the simulator. I will ask you questions about each step of the VP. Please answer these questions as precisely and with as much detail as possible. I will record this interview in order to analyse it later on. Are you okay with this? Do you have any questions so far?

Sub Steps:

Move patient to OR table - Locate entry point - Surgical scrub and covering of the patient - Team Time Out – Skin incision (optional) - Insertion of the trocar - Inject cement - Remove the trocar - Skin suture and final C-Arm image control

Questions for every Sub Step:

- *objectives/sequence of actions*
    - What are the objectives for this sub step?
    - How do you proceed?
- *responsibility*
    - Who is responsible?
- *decisions/base for decisions*
    - What decisions do you need to make?
    - On what basis do you make these decisions?
- *attention*
    - What do you focus your attention on?
- *information*
    - Which information is important?
    - Where do get this information from?
    - Do you use any imaging (CT/fluoroscopy)?
- *feedback/sensory experience* – What feedback do you get through…
        - sensory experience?
        - auditory experience?

        - visual experience?
        - other team members?
- *equipment and materials*
    - What tools and equipment do you use?
- *communication*
    - What communication is necessary?
        - With colleagues of the same profession (assistant/senior)?
        - With nurse?
        - With anesthetists?
        - With patient?
- *coordination within the team - What coordination takes place?*
    - Who performs the preliminary work for you, who supports you?
    - Are other team members dependent on your work? Do you do the preliminary work for someone else?
- *time-sensitive steps*
    - Is this sub step time-sensitive?
- *automated action*
    - Is this action automated?
- *potential complications*
    - What kind of complications could occur?
- *variations*
    - Are there any variations to your approach?
    - Is it possible to leave out this sub step?
